# Stimulus onset predictability modulates proactive action control in a Go/No-go task

**DOI:** 10.3389/fnbeh.2015.00101

**Published:** 2015-04-27

**Authors:** Marika Berchicci, Giuliana Lucci, Donatella Spinelli, Francesco Di Russo

**Affiliations:** ^1^Department of Movement, Human and Health Sciences, University of Rome “Foro Italico”Rome, Italy; ^2^IRCCS Santa Lucia FoundationRome, Italy; ^3^Department of Psychology, University of Rome ‘La Sapienza’Rome, Italy

**Keywords:** event-related potentials, prefrontal cortex, Go/No-go, timing, proactive control

## Abstract

The aim of the study was to evaluate whether the presence/absence of visual cues specifying the onset of an upcoming, action-related stimulus modulates pre-stimulus brain activity, associated with the proactive control of goal-directed actions. To this aim we asked 12 subjects to perform an equal probability Go/No-go task with four stimulus configurations in two conditions: (1) uncued, i.e., without any external information about the timing of stimulus onset; and (2) cued, i.e., with external visual cues providing precise information about the timing of stimulus onset. During task both behavioral performance and event-related potentials (ERPs) were recorded. Behavioral results showed faster response times in the cued than uncued condition, confirming existing literature. ERPs showed novel results in the proactive control stage, that started about 1 s before the motor response. We observed a slow rising prefrontal positive activity, more pronounced in the cued than the uncued condition. Further, also pre-stimulus activity of premotor areas was larger in cued than uncued condition. In the post-stimulus period, the P3 amplitude was enhanced when the time of stimulus onset was externally driven, confirming that external cueing enhances processing of stimulus evaluation and response monitoring. Our results suggest that different pre-stimulus processing come into play in the two conditions. We hypothesize that the large prefrontal and premotor activities recorded with external visual cues index the monitoring of the external stimuli in order to finely regulate the action.

## Introduction

Timing is critical for action. A planned action, such as pressing the car accelerator at the traffic light, is appropriate only if the execution is synchronized to the critical external event (i.e., light turning to green); we have to inhibit the planned action until the appropriate signal appears (Jaffard et al., 2008; Criaud et al., 2012), at the same time evaluating the elapsed time in order to anticipate the salient event. Action planning, inhibition and time processing are closely intertwined in the period before action onset. In the present study we focused on the cortical activities present during this temporal window using a Go/No-go task, which simulate a context similar to the everyday life experience above described.

According to Coull and Nobre (2008), timing tasks can be broadly divided into explicit or implicit tasks. Explicit tasks such as temporal discrimination of duration or temporal reproduction, in which the subjects use a temporal template to judge the current elapsed duration and signal when the stored interval is elapsed; these tasks activate a distributed right-lateralized frontal-striatal network (Coull et al., 2013), including the supplementary motor area (SMA), bilateral frontal-parietal regions (Lewis and Miall, 2003; Bueti et al., 2010), and the inferior Frontal gyrus (iFg; Wiener et al., 2010). More relevant for the present study is implicit temporal processing, that involves a partially different network. Implicit temporal prediction studies have used various experimental paradigms; the main difference between these paradigms is whether temporal expectation is exogenous- or endogenous-based. In *exogenous* (or externally-driven) tasks, sensory cues provide information about the onset of a task-relevant stimulus, allowing pre-allocation of cognitive resources at a specific time (called “temporal orientation of attention”). In *endogenous* (or internally-driven) tasks, also called “foreperiod” tasks because of a warning followed by an imperative stimulus, the information about the onset is not available, and the unidirectional nature of the elapsing time intrinsically biases the target predictability of stimulus occurrence over time (Coull and Nobre, 2008; Vallesi, 2010; van Rijn et al., 2011; Coull et al., 2013; Mento et al., 2015). In some studies the endogenous tasks are. Exogenous tasks entail a left-lateralized cortical circuit, including inferior parietal cortex, premotor cortex and SMA (Macar et al., 2006; Davranche et al., 2011; Coull et al., 2013). Endogenous tasks involve left inferior parietal cortex and cerebellum (Coull and Nobre, 1998), ventral premotor cortex (Coull et al., 2000), but also the right lateral prefrontal cortex (PFC; Stuss et al., 2005; Vallesi et al., 2009) and bilateral PFC (Mento et al., 2013, 2015). The cortical mechanisms underlying implicit timing are not always consistent across studies suggesting that the specificity of the task administered and the experimental context might have relevant effects (Coull et al., 2013). This inconsistency encourages the adoption of new experimental conditions, as Go/No-go tasks, which were only rarely used (Cui et al., 2009). In this latter case, in addition to SMA, also superior temporal gyrus (STG) activity was recorded. The use of event-related potential (ERP) technique with its fine temporal resolution, allows separating pre- and post-stimulus activities, which overlap in most fMRI studies, except for the study published by Cui et al. (2009), which was event-related and distinguished between pre- andpost-stimulus phase.

In the present study, we compared two conditions of implicit timing. In the “cued” condition, the time of stimulus onset was provided by informative visual cues (i.e., exogenous-based). Previous literature on the external cues demonstrated that cues orient attention in time, facilitating reaction (Niemi and Näätänen, 1981; Coull and Nobre, 1998). However, differently from other studies in which the cue informs on whether the interval is long or short (e.g., Coull et al., 2013), our cue was much more informative, i.e., progressive motion of the cues from periphery to central fixation point indicated the exact moment of stimulus onset. In this way, subjects had to synchronize their action with the end of this visual motion; without any additional task, i.e., they had not to remember any interval. This type of task is not far from the “counting down” condition used by Cui et al. (2009), although time intervals are very different (much longer in their study). In the “uncued” condition, the time of stimulus onset was not predicted by external information. Thus, in order to respond as fast as possible, one has to predict the time of stimulus onset, and the longer waiting, the greater is the probability that the event will occur at the next moment (according to the “hazard function”; see Niemi and Näätänen, 1981). In other terms, the present uncued condition was internally driven (or endogenous-based). However, differently from previous studies (Vallesi et al., 2008; Cui et al., 2009; Coull et al., 2013; Mento et al., 2013, 2015), the stimulus was not preceded by any warning. There was not a beginning of the trial (apart from the very first trial).

In brief, we used a Go/No-go task, in which subjects had to press the button in case of Go stimuli and to refrain from pressing in case of No-go stimuli. After any Go or No-go stimulus subjects had to wait for a new stimulus. The inter-stimulus interval (ISI) was held constant and the subject was not informed about the ISI duration.

ERP literature about Go/No-go task showed two main scalp activities characterizing the pre-stimulus temporal window. The cingulate motor area (CMA) and SMA are the sources of the well-known Bereitschaftspotential (BP component), representing the action preparation (for a review, see Shibasaki and Hallett, 2006) up to the decision to act (Fried et al., 2011); the PFC is the source of an even earlier slow-rising negative wave responsible for proactive action control, possibly by inhibiting the CMA-SMA (Jaffard et al., 2008; Berchicci et al., 2013; Perri et al., 2014a,b, 2015). Both activities might also reflect the temporal processing involved in the Go/No-go task. The view that motor areas may have such a role is supported by studies using warning, which interpreted the contingent negative variation (CNV) as general index of non-specific preparation and reliable hallmark of temporal prediction processing (Miniussi et al., 1999; Los and Heslenfeld, 2005; van Rijn et al., 2011; Mento et al., 2013, 2015). Support to the view that PFC might have a role in temporal processing comes from the proposal of PFC as a braking mechanism that locks the movement until the appropriate time for motor response is reached (namely, proactive inhibitory control; Jaffard et al., 2007, 2008; Aron, 2011; Berchicci et al., 2012; Criaud et al., 2012; Di Russo et al., 2013a). The focus of works on Go/No-go task is generally on inhibition; however, also the temporal aspect is clearly into play. On the one hand, inhibition is temporally limited up to the response time; on the other hand, the PFC activation was shown in some implicit (Stuss et al., 2005; Vallesi et al., 2007, 2009; Wiener et al., 2010; Mento et al., 2015) and explicit (Rao et al., 2001; Lewis and Miall, 2003, 2006) time-related tasks. It is possible that also other cortical areas are active during the pre-stimulus phase. Using fixed- and variable-foreperiod paradigms, the right-DLPFC was identified as a key region in the monitoring of temporal information (Vallesi et al., 2007, 2008); further, some neuroimaging studies (Rao et al., 2001) suggested that in addition to the PFC also inferior parietal cortex and the basal ganglia were crucially involved, whereas others (Cui et al., 2009) showed the contribution of STG in addition to SMA.

Taking into account the complexity of the results above summarized, it is interesting to evaluate whether the selected experimental conditions, which share some aspects with previous investigations (Libet, 1985; Coull et al., 2013; Mento et al., 2015), but have also different characteristics (see in particular the externally-driven condition), and refer to a different context (the Go/No-go task) are consistent with previous findings. We expect to record PFC and premotor area activities in the temporal window preceding stimulus and action, and that the difference between cued and uncued condition may be expressed in terms of strength of activation of the same areas and/or involvement of additional cortical areas. Following CNV data (Miniussi et al., 1999; Los and Heslenfeld, 2005; van Rijn et al., 2011; Mento et al., 2013, 2015), temporal orienting of attention (present cued condition) should improve the cognitive and motor preparation to the stimulus onset, with larger activity in the motor-related regions (SMA and CMA); we also expect modulation of the activity in prefrontal areas responsible for proactive action control. The DLPF area (Vallesi et al., 2007, 2008), the inferior parietal cortex (Rao et al., 2001) or the STG (Cui et al., 2009) might be additionally recruited.

The paper presents two additional results with respect to those introduced above. First, since the ISI was held constant in the abovementioned conditions, the effect of a variable ISI (using the uncued task) with reduced stimulus predictability was evaluated as a control experiment. Second, although we mainly focused on pre-stimulus phase, also data on post-stimulus phase are reported. Finally, the effect of temporal orienting, i.e., cued condition, should produce more accurate performance and faster RTs (Niemi and Näätänen, 1981; Coull and Nobre, 1998) than the uncued condition, and possibly should modulate some, relatively late, ERPs components (i.e., the P3 component) as suggested by few studies (for a review, see Correa et al., 2005, 2006a).

## Materials and Methods

### Participants

Twelve healthy participants (mean age 21.9 ± 2.4 SD years, five female) took part in the present experiment participating to both cued and uncued conditions. As control experiment, a different group of subjects (*N* = 12; mean age 24 ± 2.3 SD years) performed the uncued task, but with variable ISI. Given the methodological limitations in applying a between-subjects experimental design and the known intra-subjects variability, the results of this latter group were not submitted to statistical analyses; their data are presented in figure only for comparisons purposes in the Discussion section. All of the participants (university students) had normal vision and were fully right-handed (Edinburgh handedness inventory; Oldfield, 1971). The experimental protocol was approved by the local ethical committee and was performed in accordance with the declaration of Helsinki. All of the participants gave written informed consent.

### Apparatus and Procedure

The participants were individually tested after a 64-channel electroencephalographic (EEG) active-cap was mounted on their scalp; they were seated in a darkened room in front of a screen placed 114 cm from their eyes. A yellow point (0.15° × 0.15° of visual angle) placed at the center of the screen was used as a fixation point and was always present on the screen.

**Figure 1:**
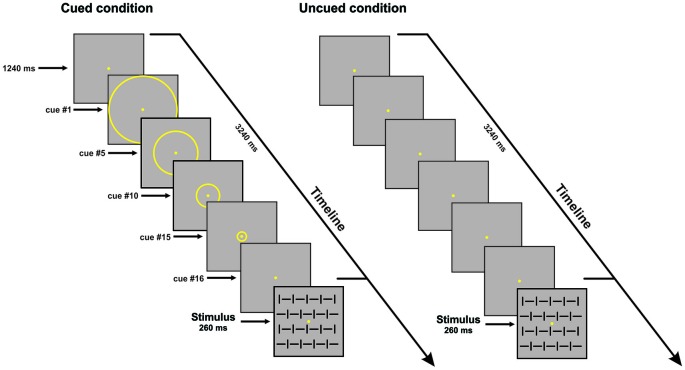
**Schematic illustration of the time sequence of events in one trial for the cued (left) and uncued (right) conditions**. For both conditions one of the four stimulus configurations is shown; stimulus duration was 260 ms. In the cued condition, 16 circles of progressively decreasing diameter were displayed (125 ms each; overall 2 s); only few examples of these circles are shown in the figure; the subject perceived centripetal motion. inter-stimulus interval (ISI) is 3.5 s. Uncued condition: only the fixation point was displayed. ISI is 3.5 s.

The visual stimuli consisted of four squared configurations made by vertical bars, horizontal bars, and both of them at the same time with different orientation (vertical and horizontal) subtending 4 × 4° presented centrally on a dark gray background; two configurations were defined as targets (go stimuli), and two were defined as non-targets (No-go stimuli). The stimuli were randomly displayed for 260 ms with equal probability (*p* = 0.25) and behavioral data were acquired using Presentation™. The presentation order of the stimuli was randomized within all blocks; ten runs allowed us to obtain a total of 800 trials (400 Go and 400 No-go stimuli). Participants were asked to respond as fast as possible to the go stimuli (*p* = 0.50) by pressing a button with their right index finger, and withhold the response when the No-go stimuli (*p* = 0.50) appeared, while maintaining high accuracy i.e., avoiding anticipation, false alarms and missing errors. The participants performed the conditions (uncued and cued) in two separate sessions; the order of conditions was counterbalanced across participants. In both cued and uncued condition, either a Go or No-go stimulus could be displayed, lasting for 260 ms. The ISI was 3.5 s in the uncued condition. In the cued condition, before stimulus, a sequence of yellow concentric circles (*N* = 16) with progressively smaller diameters (from 3.75° to 0.15°) was displayed on the screen. Each circle was displayed for 125 ms; the subject perceived a peripheral circle moving towards the fixation point. After the offset of the smallest circle, the stimulus was displayed. The apparent motion toward the center, lasting overall 2 s, cued the timing of the stimulus onset, which was certain. The ISI was 3.5 s (see Figure 1). For the variable ISI uncued control experiment, the ISI ranged from 1 to 2 s.

### Behavioral Data Analysis

The accuracy was measured by the percentage of omissions (missed responses or responses longer than 1000 ms) and false alarms (responses to No-go stimuli). The median response times (RTs) for correct trials was calculated for each participant; the median was used because the mean RTs distributions are usually positively skewed, therefore the median is the appropriate measure (Baayen and Milin, 2010). The mean RT and its standard deviation (SD) were used to calculate the intra-individual coefficient of variation (ICV = SD/mean RT). Paired-samples *t*-tests were separately performed for each behavioral measure between conditions (cued vs. uncued). The overall alpha level was fixed at 0.05.

### Electrophysiological Recording and Analysis

The continuous EEG was recorded using the BrainVision™ system with 64 active (ActiCap™) electrodes (BrainProducts GmbH., Munich, Germany) mounted according to the 10-10 International System, which were referenced to the left mastoid. The EEG was digitized at 250 Hz, amplified (bandpass of 0.01–80 Hz, including a 50 Hz notch filter) and stored for off-line averaging. Horizontal eyes movements (electrooculogram, EOG) were monitored with bipolar recordings from electrodes at the left and right outer canthi. The blinks and vertical eyes movements were recorded with electrodes below and above the left eye. Offline analysis was performed utilizing the BrainVision™ Analyzer 2.0.1 software (Brain Products GmbH., Munich, Germany).

To examine the brain activity related to both response preparation and stimulus perception, EEG recordings were separately segmented and averaged into non-overlapping 2000-ms epochs that were measured from 1100 ms before to 900 ms after the stimulus onset. Although the pre-stimulus activity would be similar for both Go and No-go stimuli, post-stimulus activity would not; thus, all analyses were performed on the Go trials only, because the study aims at investigating both pre- and post-stimulus brain activity with the same segmentation. Raw EEG data were visually inspected to identify and discard epochs contaminated with artifacts prior to the signal averaging. The first trial of each block was discarded from further analysis. The trials with artifacts (e.g., blinks or gross movements) and amplitude exceeding the threshold of ±110 μV were automatically excluded from the averaging, whereas eyes movements’ artifacts were corrected using the Gratton et al. (1983) algorithm. To further reduce high-frequency noise, the time-locked EEG grand-averages were band-pass filtered using an IIR filter (0.01–25 Hz; 24 dB/oct). The baseline was derived from the mean amplitude over the initial 200 ms of the averaged epochs.

The mean amplitude in the −500/0 ms time window, reflecting activity during the preparation stage, was selected for further analysis over Fp1 and Fp2, roughly overlaying the iFg and the anterior Insula (aIns) according to a previous fMRI study (Di Russo et al., 2013b). The mean amplitude in the −300 + 100 ms time window over FCz and Cz, roughly overlaying SMA and CMA, was used for the analysis of the BP, reflecting brain activity during motor planning (Di Russo et al., 2013b).

The amplitude of the post-stimulus prefrontal positivity (pP) was calculated peak-to-peak using as reference the preceding negative peak over prefrontal sites (Fp1 and Fp2). The pP latency was calculated on the maximum peak. Peak amplitudes and latencies of other post-stimulus ERP components were calculated for each participant in the following time windows: P1: 80–150 ms, N1: 130–200 ms, N2: 180–300 ms, and P3: 350–600 ms. The electrodes selection was based on both the scalp topography, which allowed identification of the greatest activity for a given component at the group level i.e., PO7 or PO8 for the P1 and N1 components; FCz and Cz for the N2 component; Pz for the P3 component, and previous reports (e.g., Di Russo et al., 2002; Shibasaki and Hallett, 2006; Berchicci et al., 2012).

To statistically evaluate the time windows in which the averaged activity was different from baseline signal, a *t*-test against zero (baseline) was preliminary performed for the relevant electrodes. Afterwards, the mean activity before stimulus and movement onset was submitted to four separate repeated-measures analyses of variance (RM-ANOVAs) with Condition (cued vs. uncued) as predictor factors and Sites (Fp1 vs. Fp2 for the prefrontal activities before and after stimulus-onset, FCz vs. Cz for the BP and N2 components) as repeated factors. *Post hoc* comparisons were conducted using Bonferroni correction. Furthermore, peak latency and amplitude for the P1, N1, and P3 components were separately submitted to a paired-samples *t*-tests between conditions (cued vs. uncued). The overall alpha level was fixed at 0.05.

To visualize the voltage topography of the ERP components, spline interpolated 3-D voltage maps were constructed using the BESA 2000 software (MEGIS Software GmbH, Gräfelfing, Germany). Furthermore, to investigate more deeply the pre-stimulus activity, current source density (CSD) maps were obtained using the same software. The CSD transform acts as a spatial filter and provides an estimate of the local radial current density and represents components of the primary neural activity in the scalp region (see Hjorth, 1991; Nunez and Pilgreen, 1991 for more information).

## Results

### Behavioral Data

The participants were significantly (*t*_11_ = −3.646, *p* = 0.003) faster in the cued (433 ± 56.4 ms) than uncued (480 ± 82.2 ms) task. The ICV was comparable in the two conditions (0.17), as well as the accuracy (False alarms: 6.19% and 7%; Omissions: 1.25% and 1.8% in the cued and uncued conditions, respectively).

### Electrophysiological Data

**Figure 2:**
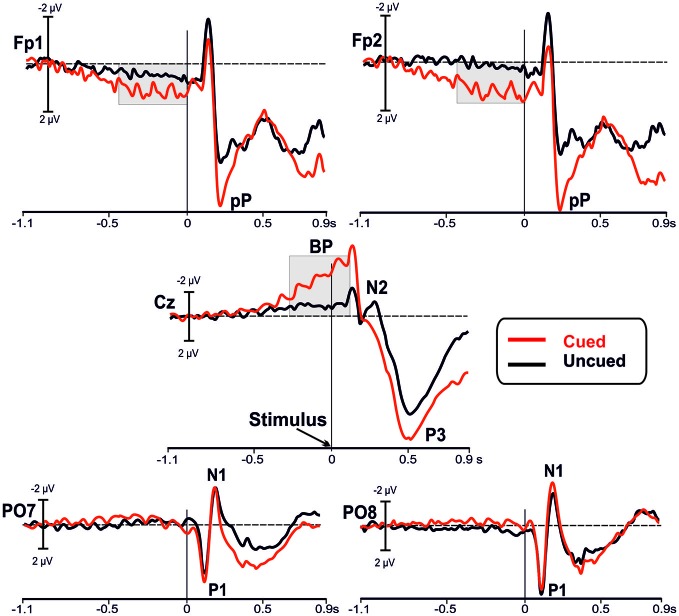
**Grand averaged waveforms of the event-related potentials (ERPs) in the in the bilateral prefrontal (Fp1, Fp2), medial central (Cz), and bilateral parietal-occipital (PO7, PO8) electrodes**. The waveforms from the uncued (black lines) and the cued (red lines) conditions are superimposed. The main components are labeled in the figures: Bereitschaftspotential (BP); prefrontal positivity (pP); P1, N1 and P3 components.

Figure 2 shows the grand-averaged waveforms of the ERPs for both cued (red lines) and uncued (black lines) tasks at bilateral prefrontal (Fp1 and Fp2), medial central (Cz) and bilateral parietal-occipital (PO7 and PO8) sites.

#### Before Stimulus Onset

Inspection of the figure shows that a sustained prefrontal positive activity was present at the prefrontal leads (Fp1 and Fp2) in the cued condition before stimulus onset; this activity was much lower in the uncued condition. The slow rising negative BP component at central site (Cz) was present in both tasks, even though it was larger in the cued than uncued condition. In this time window, we did not observe other relevant activities. Statistical analysis (RM-ANOVA) of the mean amplitude over prefrontal regions before stimulus onset showed a main effect of Condition (*F*_1,22_ = 7.853, *p* = 0.010), with larger amplitude in the cued (2.00 ± 0.4 μV) compared to uncued (0.68 ± 0.3 μV) condition. The ANOVA did not reveal a significant Site effect (*F*_1,22_ = 0.014, *p* > 0.9) or Condition × Site interaction (*F*_1,22_ = 0.461, *p* > 0.5). Statistical analysis of the BP mean amplitude did yield a significant result as a function of Condition (*F*_1,22_ = 46.72, *p* < 0.0001), with larger amplitude in cued (−4.93 ± 1.9 μV) than uncued (−0.96 ± 1.0 μV) condition. The effect of Site (*F*_1,22_ = 0.01, *p* > 0.9), and Condition × Site interaction (*F*_1,22_ = 0.461, *p* > 0.5) were not significant.

#### After Stimulus Onset

After stimulus onset, over parietal-occipital sites the visual P1 and N1 components are observed at the expected latencies (116 and 183 ms, respectively) and were nearly overlapped in the two conditions. Peaking at around 200 ms at Fp1 and Fp2 electrodes, the pP component was clearly detectable and, even though its peak amplitude was larger in the cued condition, its peak-to-peak amplitude was not so different from pP of the uncued condition. In addition, the N2 component recorded at central site (Cz) was clearly present in the uncued condition only. In contrast, the P3 component was larger in the cued than uncued condition.

Statistical analysis of the P1 did not show significant differences between conditions (all *p*s > 0.9) neither for latency nor amplitude; same result holds true for the N1 (all *p*s > 0.9). The pP latency and peak-to-peak amplitude were not affected by condition (all *p*s > 0.5). Analysis of the N2 did not yield significant results (all *p*s > 0.20). Analysis of P3 latency did not reach the significance threshold (*t*_11_ = 2.063, *p* = 0.060), even though there was a tendency to peak earlier in the cued (491 ± 52 ms) than uncued (526 ± 57 ms) condition. Finally, the P3 amplitude was significantly larger (*t*_11_ = −2.300, *p* = 0.042) in the cued (17.9 ± 5.5 μV) than uncued (14.1 ± 6 μV) condition.

### Topographical Mapping

**Figure 3:**
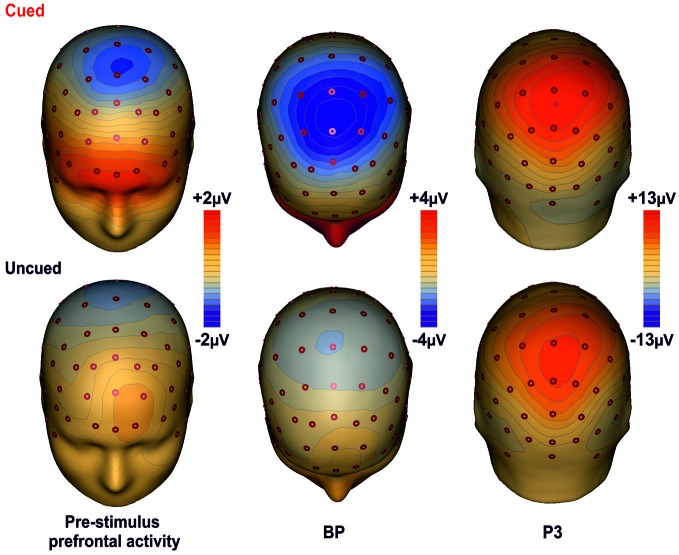
**Topographical maps of the ERP components**. The maps for the cued task are shown on the first row; the second row shows the maps for the uncued task. The maps report the main cortical activities in different time intervals: Prefrontal activity in the interval −400/0 ms; BP component in the −300/+100 ms time interval; P3 component in the interval 350–600 ms.

**Figure 4:**
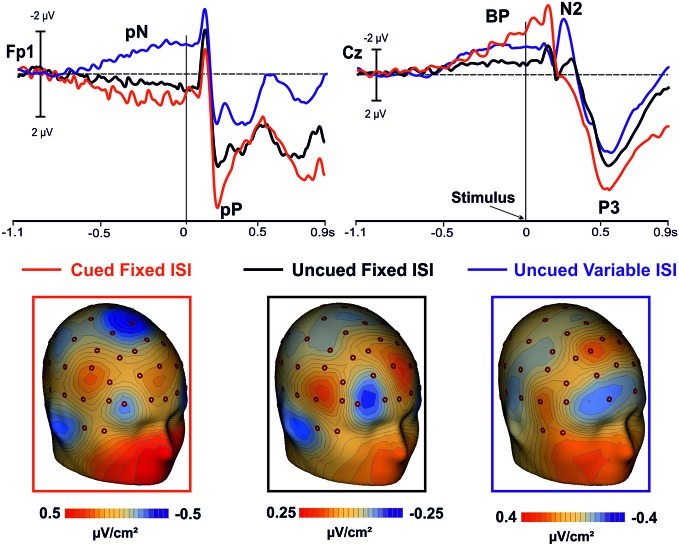
**In the top panel, grand averaged waveforms of the ERPs in the prefrontal (Fp1), and medial central (Cz) sites for the cued-fixed ISI (red lines), uncued-fixed ISI (black lines)**. Data from a similar uncued task but with variable ISI (blue lines) are also shown. In the lower panels, current source density (CSD) maps of the pre-stimulus activity (300/0 ms) over prefrontal regions are displayed.

Figure 3 shows the scalp topographies of the relevant ERPs components for the cued (top row) and the uncued (bottom row) condition. The maps are displayed from the left to the right according to the timing of each component. In the first column maps, a wide spread positivity is observed over prefrontal regions, bilaterally distributed in the cued condition and in the left hemisphere for the uncued condition. Although this difference in the scalp voltage distribution, the CSD maps displayed in Figure 4 show that the source of the positivity could be located in similar bilateral prefrontal regions over the iFg. The maps in the second column show the negativity in premotor areas (as indexed by the BP), which is less anterior in the uncued than cued task. The maps of the last column show the topography of the P3, a positive distribution over medial central-parietal areas, which did not differ in topography between conditions.

## Discussion

The present study investigated electro-cortical and behavioral responses during a discriminative visuo-motor task in which the temporal predictability of the stimulus appearance was manipulated by the presence/absence of exogenous temporal informative cues.

Behavioral data confirm numerous previous studies (Coull and Nobre, 1998; Stuss et al., 2005; Vallesi et al., 2007, 2009; Mento et al., 2015; see Niemi and Näätänen, 1981 for a review) and show that temporal orientation (cued condition) allows faster motor response to targets, even though accuracy was comparable (Davranche et al., 2011). However, the accuracy in both cued and uncued conditions was very high; thus, the lack of accuracy improvement could be due to a ceiling effect.

At cortical level, the most interesting results concern the pre-stimulus phase. Scalp recorded activity on prefrontal and premotor regions was much larger in the cued than uncued condition. In the cued condition (in which the onset of the stimulus was based on exogenous information), prefrontal and premotor activities were similar in terms of polarity and localization to those recorded in the uncued condition (which required endogenous time prediction), but much larger in amplitude. Such increased activity is likely due to the monitoring of the external stimuli, which finely regulated the action. In the cued condition, the motion of the circle toward the foveal fixation point provided precise and direct information about the timing of stimulus onset. The participants observed this centripetal motion and synchronized their response on the optical flow. This processing on the one hand shorten the RTs, and on the other hand has a neural cost, as reflected by the large activities at prefrontal and premotor levels.

The positive activity recorded over the PFC gradually increased as function of time, lasted until stimulus offset, and anticipated the activity in premotor areas, which followed a pattern similar to that of the PFC. Earlier activity of the PFC with respect to the premotor regions has already been reported in studies using similar tasks (see also Berchicci et al., 2012, 2014; Perri et al., 2014a,b, 2015; Di Russo et al., 2013b), suggesting that the prefrontal cortex (in particular the iFg) exerts a sort of cognitive control before, and possibly over, the motor preparation activity. This idea is also supported by the inspection of the grand-averaged ERP waveforms collected from a different group of subjects performing an uncued task (endogenous time prediction), but with *variable* ISI, therefore, with more difficult stimulus prediction (see Figure 4 blue line). Indeed, also in this case the activity starts earlier in the PFC than premotor areas. Thus, the onset time of the PFC activity seems independent from fixed or variable ISI, and from endogenous or exogenous time processing.

It is worth of note that in the uncued condition with variable ISI the PFC activity had negative polarity while the polarity was positive in the *fixed* ISI conditions. However, looking at the CSD maps (Figure 4) it seems that the intracranial electrical dipoles locations generating the waveforms in the three conditions are quite similar and compatible with activity from bilateral iFg (based on the ERP-fMRI data of Di Russo et al., 2013b), but from different sub-regions. The main difference between conditions seems due to the dipole orientation, generated by the sub-regions within the cortex convolution of lateral fissure (were the pars opercularis of the iFg is located). Changes in ERP source orientation but not in general source location has been previously documented for the primary visual cortex in the Calcarine fissure (e.g., Di Russo et al., 2002) and justified by the high convolution of this area. The same could apply for the pars opercularis of the iFg, which is an ascending ramus within the highly convolute lateral fissure (Di Russo et al., 2013b). Further, the absolute amplitude of the uncued variable ISI was larger than the uncued fixed ISI. Future fMRI research will evaluate whether the presence of stimulus predicting visual cues modulated the iFg activity.

The BP component had the same polarity in all conditions (i.e., cued fixed-ISI, uncued fixed-ISI, and uncued variable-ISI); its amplitude seems to vary according to the amount of processing involved, which appear highest when synchronization with external stimuli was required. This latter condition (simulated by the present cued condition) is probably one of the most basic experience of the timing regulation of action (hit or avoid a ball that is approaching; grab a flying fly, etc.). However, also expectations about the possible, uncertain onset of future event is important for action, and we are able to extract this temporal information based on probabilistic temporal structure of events (Nobre et al., 2007). Support to present results comes from CNV studies, which showed larger CNV amplitude following informative than neutral cues (Miniussi et al., 1999; van Rijn et al., 2011; Mento et al., 2015). Together, these data support the view that the CNV could represent an electrophysiological index of temporal preparation for the processing of upcoming events (van Rijn et al., 2011), instead of a pacemaker-accumulator or source of temporal information (Macar and Vidal, 2003; Meck et al., 2008).

Overall, both the PFC and the premotor areas were active during the pre-stimulus phase of the present Go/No-go task and, at least in the various conditions here considered, the involvement of additional areas such as parietal (Bueti et al., 2010), DLPC (Vallesi et al., 2007, 2009) or temporal gyrus (Cui et al., 2009) was not necessary.

Regarding the post-stimulus processing, the P3 amplitude was larger when the timing of stimulus onset was predictable; these data support the view that temporal cueing may produce cognitive benefits (for a review, see Correa et al., 2006a), also evident at behavioral level. The P3 component reflects multiple processing including stimulus categorization (Dien et al., 2004) and response-related processing (Verleger et al., 2005, 2006), and larger P3 amplitudes are considered index of more in-depth cognitive processing. Modulation of the P3 component amplitude by predictable stimulus onset were previously reported with a different paradigm (Miniussi et al., 1999; Correa et al., 2006b). Overall, it is likely that predictable time of stimulus onset maximizes endogenous visual attention toward the task-relevant stimulus facilitating the subsequent link between temporal and motor processing (Rosenbaum and Collyer, 1998). The P1, N1, pP and N2 were not modulated by condition, hence the stimulus predictability does not affect early visual processing (as reflected by the P1 and N1) and stimulus-response mapping reflected by the pP (Perri et al., 2014a). The lack of effect on the N2 is likely due to the combined effect of premotor, prefrontal and parietal activities present in this time range (for a detailed interpretation of the N2 origin please see Di Russo et al., 2013b).

It is worth to mention that various studies supported the key role of the PFC in high level planning and organization of behavior (i.e., monitoring of stimulus selection from a set or occurrence of stimuli from an expected set), as well as in other cognitive processes, such as working memory updating, decision making, and many other functions (see Petrides, 2005 for a review). Moreover, several cognitive processes such as working and long term memory, decision making, inhibition and attention are usually involved in timing tasks. Thus, prefrontal and premotor regions take part in a complex timing system including proactive inhibition, decision-making, attention and working-memory processes (Wittmann, 2009). Further, neuroimaging studies with patients (Harrington et al., 1998, 2004; Koch et al., 2004) and healthy participants (Rao et al., 2001) generally agree on the hypothesis that a distribute dopamine-dependent fronto-striatal loop (for a review see Matell and Meck, 2004), the cerebellum and the posterior parietal cortex are critical for the processing of timing. In the present study, the activity began in prefrontal brain regions about 800 ms before the stimulus onset; the motor-related areas started their activity a few hundred of milliseconds later and were active up to the motor response. Moreover, the parietal regions were activated concomitantly to the response onset. ERP recording might contribute to evaluate the timing of the different processing involved in considered time prediction tasks.

## Author Contribution

The study presented here has been conceived and designed by MB, GL, DS and FDR. The experiments were performed by MB and GL. Data were analyzed by MB, GL, and FDR. Data have been interpreted by MB, GL, DS and FDR. The manuscript was written, revised and approved by MB, GL, DS and FDR.

## Conflict of Interest Statement

The authors declare that the research was conducted in the absence of any commercial or financial relationships that could be construed as a potential conflict of interest.
